# Enhanced Ethanol Sensing Performance and Humidity Tolerance of Ce/ZnO-Incorporated In_2_O_3_ Nanocubes

**DOI:** 10.3390/mi17050539

**Published:** 2026-04-28

**Authors:** Yijun Yang, Dong Geon Jung, Daewoong Jung

**Affiliations:** 1 Mobility Robot System R&D Group, Korea Institute of Industrial Technology (KITECH), Daegu 42994, Republic of Korea; yjyang@kitech.re.kr (Y.Y.); jdg8609@kitech.re.kr (D.G.J.); 2 School of Electronic and Electrical Engineering, College of IT Engineering, Kyungpook National University, 80 Daehakro, Daegu 41566, Republic of Korea; 3 Department of Nanomechatronics Engineering, College of Nanoscience & Nanotechnology, Pusan National University, 2 Busandaehak-ro, Busan 46241, Republic of Korea; 4 School of Transdisciplinary Engineering, College of Engineering, Pusan National University, 2 Busandaehak-ro, Busan 46241, Republic of Korea

**Keywords:** gas sensor, In_2_O_3_, ZnO, Ce, PCA

## Abstract

This work presents the design and evaluation of cerium and zinc oxide-incorporated indium oxide (Ce/ZnO-In_2_O_3_) nanocube composites synthesized via a hydrothermal process for advanced ethanol gas sensing. The incorporation of Ce and ZnO effectively modified the surface chemistry and electronic structure of In_2_O_3_ without causing significant morphological degradation. Compared with pristine In_2_O_3_, the Ce/ZnO-In_2_O_3_ sensor exhibited a significantly enhanced response of 33.2 toward 100 ppm ethanol at 300 °C, corresponding to an 8.7-fold improvement, along with a low detection limit of 0.8 ppm. In addition, the composite sensor demonstrated stable and reversible sensing behavior, excellent repeatability over 100 cycles, and long-term operational stability. Notably, improved humidity tolerance was achieved, with approximately 77% of the initial response retained at 80% relative humidity. The enhanced sensing performance is attributed to the combined effects of heterojunction formation between ZnO and In_2_O_3_ and Ce-induced lattice distortion, which promote oxygen adsorption and facilitate charge transfer during gas reactions. Principal component analysis (PCA) further confirmed the improved discrimination of ethanol against interfering gases. These results underscore the synergistic effects of Ce and ZnO incorporation in tailoring electronic structures and surface chemistry, thereby emphasizing the potential of this strategy for reliable ethanol detection in environmental and industrial applications.

## 1. Introduction

Ethanol (C_2_H_5_OH) is a widely used volatile organic compound in industrial processing, environmental monitoring, and food safety applications; however, excessive ethanol vapor poses potential risks due to its flammability and adverse effects on human health. Reliable detection of ethanol is therefore essential for safety control and process monitoring in various industrial environments [1,2,3,4]. To date, metal oxide semiconductors have been extensively utilized as chemical sensing materials due to their advantageous sensitivity and selectivity to atmospheric conditions, as well as their straightforward synthesis and application in sensor fabrication [5,6].

Gas sensors based on metal oxide semiconductor (MOS) technology have seen extensive implementation across a wide range of disciplines. ZnO [7,8,9], SnO_2_ [10,11,12], In_2_O_3_ [13,14], WO_3_ [15,16] and TiO_2_ [17,18] are commonly used for gas detection due to their distinct advantages, including their ease of fabrication, minimal energy requirements, and cost-effectiveness [19,20,21,22]. More recently, doped complex oxide semiconductors have emerged as a new class of high-performance gas-sensing materials; for example, Er-doped ZnGa_2_O_4_ ceramics have been demonstrated to act as high-temperature methane sensors for combustion monitoring applications [23]. These developments highlight the role of rare-earth incorporation in tuning the electronic and surface properties of oxide sensors, a strategy that motivates the Ce-based approach adopted in this work. Among them, zinc oxide (ZnO) demonstrates considerable promise in the domain of gas sensing, attributable to its substantial band gap of 3.37 eV, noteworthy electron mobility, and notable thermal stability [7,24]. Beyond chemiresistive sensing, ZnO has also been employed in optical-transduction gas sensors—for instance, Grigorjeva et al. demonstrated gas-sensitive luminescence of ZnO coatings obtained via plasma electrolytic oxidation [25]—further underscoring the material’s broad applicability in gas detection. Indium oxide (In_2_O_3_) finds application in gas sensors, solar cells, and photocatalysis, with the bandgap of this material ranging from 3.55 to 3.75 eV [26,27]. In the field of gas sensors, ZnO and In_2_O_3_ have been identified as a noteworthy material for gas detection [28,29], owing to their high conductivity and wide bandgap. Nevertheless, practical limitations remain, including relatively high operating temperatures, insufficient selectivity, and strong performance degradation under humid conditions, which hinder their reliable application in real-world environments.

Especially, the impact of moisture on gas sensors is an inevitable concern in practical applications and is increasingly recognized in the field of gas sensor research due to its pervasiveness and variability [30,31,32]. The presence of water vapor has been demonstrated to have a substantial impact on the baseline resistance and gas response of gas sensors and has also been shown to result in a reduction in the service life of the devices [7,33,34].

To address these challenges, various strategies have been explored, including elemental doping, heterojunction formation, and surface modification. The incorporation of rare-earth elements, such as cerium (Ce), has been reported to effectively regulate oxygen vacancy concentration and surface oxygen adsorption through reversible redox behavior (Ce^3+^/Ce^4+^), leading to improved sensing response and humidity tolerance. Specifically, Han et al. reported an about 2.8-fold improvement in methanol sensing response through Ce doping of In_2_O_3_ porous nanospheres, with Ce^3+^/Ce^4+^ coexistence and increased oxygen vacancy density confirmed [35]. Bai et al. demonstrated that Ce-doped In_2_O_3_ nanosheet-assembled hierarchical microstructures achieve an isopropanol response of ~31 at 100 ppm with a detection limit of 1.2 ppm at 220 °C, as well as Ce^3+^ fractions of ~30% [36]. Sun et al. showed enhanced formaldehyde sensing performance of MOF-derived Ce-doped hollow In_2_O_3_ nanoboxes, directly correlating the Ce^3+^ fraction with oxygen vacancy density [37]. In parallel, the construction of ZnO-In_2_O_3_ heterostructures has been demonstrated to enhance charge transfer and modulate interfacial potential barriers, thereby improving gas-sensing performance. Zhang et al. synthesized ZnO-In_2_O_3_ hierarchical heterostructures with an ethanol response of ~28.4 at 100 ppm (260 °C), attributed to the n-n heterojunction interface [29]. Wang et al. clarified that the direction of electron transfer at the ZnO/In_2_O_3_ interface governs the dominant sensing mechanism [38]. Jiang et al. further showed that mesoporous In_2_O_3_-ZnO hierarchical structures provide an ethanol response of ~22.7 at 100 ppm (280 °C) due to the enhanced specific surface area and heterojunction-modulated potential barriers at grain boundaries [39]. Despite these advances, a systematic understanding of the combined effects of rare-earth incorporation and oxide heterojunction formation on sensing behavior remains limited. In this study, Ce- and ZnO-incorporated In_2_O_3_ nanocubes were synthesized via a hydrothermal route and comparatively investigated to elucidate the roles of Ce incorporation and ZnO-In_2_O_3_ heterojunctions in ethanol sensing performance, stability, and humidity tolerance.

In the present work, In_2_O_3_ nanocubes incorporating Ce and/or ZnO were synthesized using a hydrothermal method, and their ethanol gas-sensing properties were systematically compared with those of pristine In_2_O_3_. By preparing four representative samples, pristine In_2_O_3_, Ce-In_2_O_3_, ZnO-In_2_O_3_ composites, and Ce/ZnO-incorporated In_2_O_3_, the individual and combined effects of Ce incorporation and ZnO-In_2_O_3_ heterojunction formation on sensing performance were clarified. The sensors were evaluated in terms of response magnitude, detection limit, repeatability, long-term stability, selectivity, and humidity tolerance under controlled operating conditions. The results provide a comprehensive understanding of how rare-earth incorporation and oxide hetero-interfaces collectively influence the sensing behavior of In_2_O_3_-based materials, offering insights into the design of reliable ethanol gas sensors with improved environmental robustness.

## 2. Materials and Methods

### 2.1. Synthesis of Sensing Material

Indium oxide nanocube (In_2_O_3_ NC) was synthesized via the hydrothermal method. Firstly, 50 mM of sodium carbonate (Na_2_CO_3_, 99.00%, Darmstadt, Germany, Sigma-Aldrich) and 50 mM of indium chloride (InCl_3_, 99.00%, Darmstadt, Germany, Sigma-Aldrich) were solved in 30 mL of distilled water, and the mixture solution was stirred for 10 min. Then, this obtained solution was transferred to a Teflon-lined autoclave. The synthesis reaction was conducted at 90 °C for 10 h in a box furnace. After conduction, the temperature was naturally cooled down to room temperature (about 30 °C). Finally, the yellow-colored sediment was washed several times with ethanol and water, then dried at 60 °C overnight. Finally, the obtained powder was calcined at 500 °C for 2 h in a furnace. For Ce and ZnO-composited In_2_O_3_ nanocubes, certain amounts of 10 mM of cerium (III) chloride heptahydrate (CeCl_3_∙7H_2_O, 99.90%, Darmstadt, Germany, Sigma-Aldrich) and 30 mM of zinc chloride (ZnCl_2_, 98.00%, Darmstadt, Germany, Sigma-Aldrich) were separately added to the precursor solution during stirring. To evaluate the effects of Ce and ZnO on gas-sensing performance, four types of samples were prepared: pristine In_2_O_3_, Ce-In_2_O_3_, ZnO-In_2_O_3_ and Ce and ZnO-incorporated In_2_O_3_ (Ce/ZnO-In_2_O_3_). The corresponding weight ratios of the components were 16.8 wt% Ce and 83.2 wt% In_2_O_3_ for Ce-In_2_O_3_, 26 wt% ZnO and 74 wt% In_2_O_3_ for ZnO-In_2_O_3_, and 22.6 wt% ZnO, 13 wt% Ce, and 64.4 wt% In_2_O_3_ for Ce/ZnO-In_2_O_3_, respectively. For Ce/ZnO-In_2_O_3_, a weight ratio of about 3.42:1 for Ce-In_2_O_3_ and ZnO was used for synthesizing the sensing material. The molar ratios of each component, In_2_O_3_, ZnO and Ce, were determined through preliminary research and references [38,39,40,41].

### 2.2. Fabrication of Ethanol Gas Sensor

To fabricate the In_2_O_3_ NCs ethanol gas sensor, the following process was prepared. The alumina plate was cleaned by ultrasonication using acetone, methanol and deionized water. The interdigitated electrode (IDE) was patterned with screen-printing using silver paste. Then, the In_2_O_3_, Ce-In_2_O_3_, ZnO-In_2_O_3_ and Ce/ZnO-In_2_O_3_ powders were separately stirred with a proper quantity of water to obtain a paste and screen-printed on IDE. The plate diced to a size of 5 mm × 5 mm each.

### 2.3. Material Characterization

The crystal structure of the In_2_O_3_ nanocube was analyzed by X-ray diffraction (XRD, model Empyrean, Almelo, the Netherlands, Panalytical) with Cu Kα radiation. Surface morphology analysis was carried out by field emission scanning electron microscope (FE-SEM, model SU8220, Tokyo, Japan, Hitachi). The chemical elements of each sample were analyzed by X-ray photoelectron spectroscopy (XPS, model NEXSA, Waltham, MA, USA, ThermoFisher).

### 2.4. Gas-Sensing Performance

**Figure 1:**
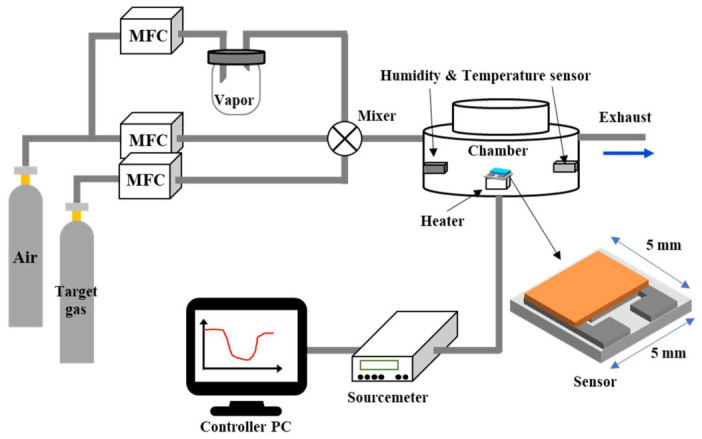
Schematic of gas-sensing setup.

The evaluation of gas-sensing performance was conducted within a manufactured gas chamber, as shown in Figure 1. The chamber was made from stainless steel with a 13 cm radius and a 18 cm high cylindrical shape. The adjustment of gas concentration was facilitated by a mass flow controller (MFC, Busan, Republic of Korea, Nextron) and the inner chamber condition was measured by a temperature and humidity sensor (BLUETEC, BO-807, Republic of Korea) during all experiments. In order to ascertain the alterations in resistance engendered by the gas flow environment, a voltage of 1 V was applied to the tested gas sensor by means of a source meter (model B2902B, Santa Rosa, CA, USA, Keysight). The measurement was performed by fixing the sum of air and target gas flow rate (a total of 1000 sccm) using MFC and adjusting the target gas concentration ratio. For this gas-sensing experiment, synthetic air (99.999%, 47 L), air-balanced ethanol (200 ppm, 10 L), air-balanced acetone (100 ppm, 10 L), air-balanced formaldehyde (100 ppm, 10 L), air-balanced methane (5000 ppm, 10 L), air-balanced ammonia (2000 ppm, 10 L) and air-balanced carbon monoxide (2000 ppm, 10 L) were purchased from Korea Standard Gas Co., Ltd. (Daegu, Republic of Korea). The response to the target gas is calculated using Equation (1), as follows:

Response = ΔR/R = |R_0_ − R_g_|/R_g_ (1)

where R_0_ is the measured sensor resistance in the air and R_g_ is the resistance value when exposed to the target gas. For In_2_O_3_, an n-type semiconductor, the resistance decreases for the reducing gas and increases for the oxidizing gas. Since all six target gases used in this study (ethanol, acetone, formaldehyde, methane, ammonia, and carbon monoxide) are reducing gases, Equation (1) was employed to calculate the sensor response. The sensor operating temperature was controlled via a resistive ceramic micro-heater mounted beneath the alumina substrate, regulated by a PID controller receiving feedback from a calibrated K-type thermocouple (wire diameter 0.2 mm, NIST-traceable) in contact with the heater–substrate interface. To ensure accurate reporting of the sensing-layer temperature, a pre-measurement calibration was performed using a secondary fine-wire thermocouple (25 μm diameter) placed directly on the sensing film surface to measure the actual layer temperature as a function of applied heater power. The resulting offset between the heater thermocouple and the sensing-film surface (typically 2–5 °C) was compensated in the PID set-point, yielding a sensing-layer temperature accuracy of ±3 °C at 300 °C. All measurements were performed only after steady-state thermal equilibrium was achieved, defined as a baseline resistance drift of less than 1% over 5 min. The humidity testing procedure was meticulously executed under controlled relative humidity (RH) in the measurement chamber, with the sensor locally heated to an elevated operating temperature. The process began by setting a dedicated humidification chamber to 70 °C, a condition under which saturated water vapor pressure was established, thereby creating the necessary wet air source as shown in Figure 1. The final humid gas mixture was precisely managed using MFC, combining the saturated H_2_O gas flow with dry air, yielding a total flow rate of 1000 sccm. The resulting relative humidity of the bulk gas inside the measurement chamber, defined at 25 °C, was monitored using a calibrated commercial RH sensor (BLUETEC, BO-807) located away from the heated sensor region to monitor the bulk gas condition. To ensure the gaseous state integrity of the mixture and prevent any condensation that could introduce measurement errors, the entire gas delivery line was rigorously maintained at a temperature between 100 °C and 110 °C using external heating tape, thereby keeping the temperature above the dew point. Concurrently, the sensor operating temperature was precisely controlled using a proportional–integral–derivative (PID) controller, which regulated the ceramic heater to maintain a stable internal chamber temperature. The gas-sensing process involved verification of the sensor temperature, relative humidity, and gas concentration. All gas-sensing experiments were conducted at an operating temperature of 300 °C and an RH of 20%, unless otherwise noted. Humidity- and temperature-dependent measurements were carried out separately. For humidity-dependent measurements (20–80% RH), the operating temperature was maintained at 300 °C, whereas temperature-dependent measurements (150 to 400 °C) were carried out under a constant relative humidity of 20% RH (defined at 25 °C).

## 3. Results and Discussions

### 3.1. Structural and Morphological Characteristics

**Figure 2:**
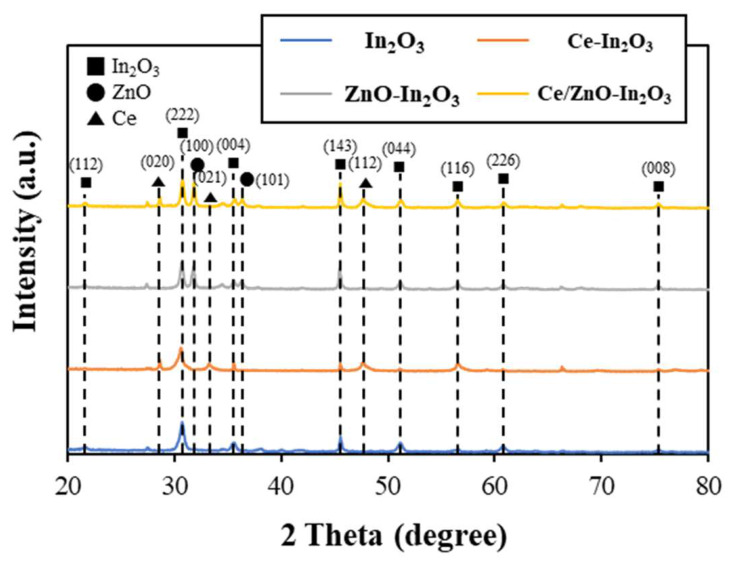
XRD of In_2_O_3_ with Ce and ZnO combined.

**Figure 3:**
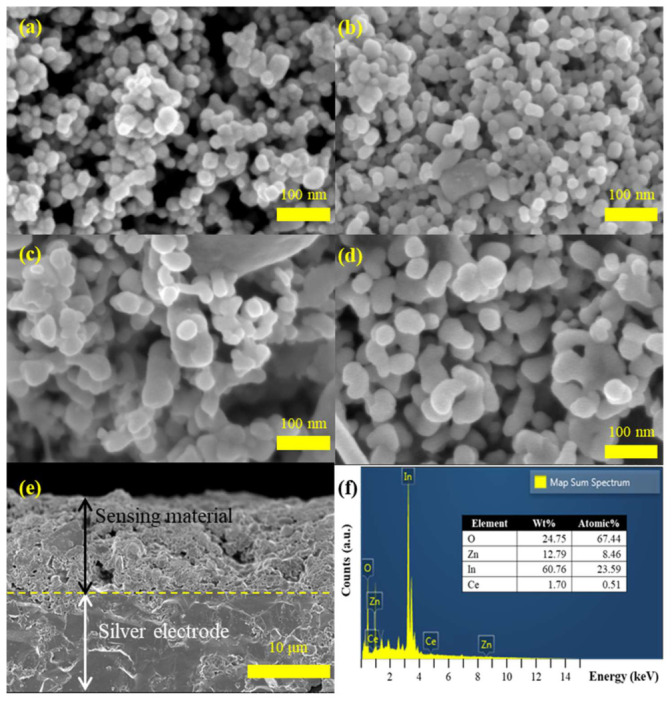
SEM top view image of (**a**) In_2_O_3_, (**b**) Ce-In_2_O_3_, (**c**) ZnO-In_2_O_3_, and (**d**) Ce/ZnO-In_2_O_3_; (**e**) cross-section image of Ce/ZnO-In_2_O_3_ and IDE electrode; (**f**) EDS spectra of Ce/ZnO-In_2_O_3_.

To investigate the microstructure of In_2_O_3_ (Ce and ZnO-incorporated) powders, X-ray diffraction (XRD) analysis was conducted. As illustrated in Figure 2, the XRD patterns and peaks of In_2_O_3_, ZnO and Ce peaks are evident. The diffraction peaks of In_2_O_3_ were observed at 21.52°, 30.62°, 35.51°, 45.75°, 51.09°, 56.06°, 60.75°, and 75.16°, corresponding to the (112), (222), (004), (143), (044), (116), (226), and (008) crystal planes of In_2_O_3_, respectively. The peaks for ZnO, which correspond to the crystal planes of (100) and (101), are seen at 31.84° and 36.33°, and peaks for Ce, corresponding to (020), (021) and (112), are also seen at 29.76°, 34.47° and 48.33°, respectively. These peaks are characteristic of the standard card of cubic In_2_O_3_ (JCPDS 06-416), hexagonal ZnO (JCPDS 89-0511) and orthorhombic Ce (JCPDS 43-0606), thereby validating the efficacy of the synthesis process for In_2_O_3_ nanocubes and substantiating the presence of the synthesized ZnO and Ce [42,43]. The d-spacings calculated from these observed 2θ values (3.000, 2.600, and 1.882 Å) match those of JCPDS 43-0606 (reference d-spacings 2.996, 2.604, and 1.881 Å) within ±0.004 Å, well within XRD instrumental uncertainty. The assignment is further supported by the following: (i) the absence of these peaks in the pristine In_2_O_3_ and ZnO-In_2_O_3_ control samples, (ii) the EDS detection of 1.70 at% Ce consistent with the observed peak intensities (Figure 3f), and (iii) the hydrothermal followed by 500 °C calcination conditions, which are known to yield mixed-valence Ce-oxide phases. The ionic radius of Ce^3+^ (~1.01 Å) is substantially larger than that of In^3+^ (~0.80 Å), a ~26% mismatch well exceeding the Hume-Rothery limit of ~15% for solid-solution formation-making complete substitutional incorporation of Ce into the In_2_O_3_ lattice, which is thermodynamically unfavorable at the Ce loading used in this study. Consequently, Ce predominantly exists as a separate orthorhombic Ce-oxide secondary phase decorating the surface and grain boundaries of the In_2_O_3_ nanocubes, rather than being uniformly substituted into the lattice. Nevertheless, interfacial Ce-In-O interactions and partial surface substitution at the phase boundary are plausible and likely contribute to the lattice distortion effects.

Figure 3 shows the morphology images of synthesized pristine NCs and NCs with Ce, ZnO-composited In_2_O_3_ by FESEM. The morphology of In_2_O_3_ nanocubes is largely similar for pristine and Ce and ZnO-incorporated ones. The pristine In_2_O_3_ nanocube shows about 15–25 nm uniform particles with smooth surfaces. After Ce is incorporated, there is no obvious change in morphological shape, but some degree of agglomeration is shown compared to pristine In_2_O_3_. Conversely, a general enhancement in the dimensions of the In_2_O_3_ nanocube is evident in the context of composited ZnO. The SEM image presents a combination of spherical and irregularly shaped particles, indicating successful incorporation of zinc oxide into the indium oxide matrix. It can be seen in Figure 3e that the thicknesses of the sensing film and IDE through screen-printing were deposited in amounts of about 12 μm for both. Additionally, Figure 3f presents energy-dispersive X-ray spectroscopy (EDS) mapping of the Ce/ZnO-In_2_O_3_. The EDS analysis confirms the coexistence of In, Zn, O and Ce elements in the composites, with the ratio of In, Zn, O and Ce being 60.76%, 12.79%, 24.75% and 1.70%, respectively.

**Figure 4:**
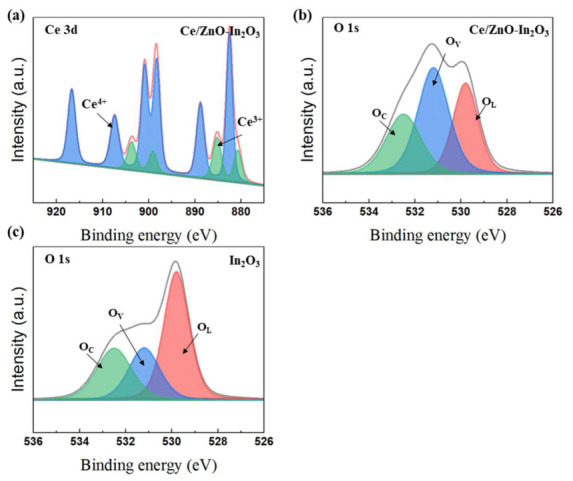
XPS spectra for (**a**) Ce 3d region of Ce/ZnO-In_2_O_3_, showing six Ce^4+^ components and four Ce^3+^ components, (**b**) O 1s region of Ce/ZnO-In_2_O_3_, showing lattice oxygen O_L_ (35.3%), oxygen vacancy O_V_ (41.3%), and chemisorbed oxygen O_C_ (23.4%), and (**c**) O 1s region of pristine In_2_O_3_ reference, with O_V_ fraction of only 22.5%.

X-ray photoelectron spectroscopy (XPS) was conducted for the Ce/ZnO-In_2_O_3_ composite and pristine In_2_O_3_ to examine the surface chemical states and to evaluate the Ce^3+^/Ce^4+^ coexistence and oxygen-vacancy formation associated with the sensing mechanism. Figure 4 presents the high-resolution Ce 3d and O 1s spectra together with the fitted components.

Figure 4a shows the Ce 3d spectrum of Ce/ZnO-In_2_O_3_. The spectrum was fitted using ten components, including six peaks assigned to Ce^4+^ and four peaks assigned to Ce^3+^, based on reported binding energies and peak-width constraints. The Ce^4+^ features include v (882.5 eV), v″ (888.8 eV), v‴ (898.3 eV), u (900.9 eV), u″ (907.4 eV), and u‴ (916.7 eV), while the Ce^3+^ contributions correspond to v_0_ (880.7 eV), v′ (885.2 eV), u_0_ (899.1 eV), and u′ (903.7 eV). The presence of both Ce^3+^- and Ce^4+^-related peaks indicates a mixed-valence Ce state in the composite. Quantitative fitting yielded a Ce^3+^ fraction of 32.4% and a Ce^4+^ fraction of 67.6%, which is comparable to values reported for related Ce-modified In_2_O_3_ sensing materials [37,43,44].

The O 1s spectra of Ce/ZnO-In_2_O_3_ and pristine In_2_O_3_ are shown in Figure 4b,c. Each spectrum was deconvoluted into three components: lattice oxygen (O_L_, 529.8 eV), defect-related oxygen species associated with oxygen vacancies (O_V_, 531.2 eV), and chemisorbed oxygen/surface hydroxyl groups (O_C_, 532.5 eV). The Ce/ZnO-In_2_O_3_ composite exhibited a significantly higher O_V_ fraction (41.3%) than pristine In_2_O_3_ (22.5%), whereas the O_L_ fraction decreased from 55.1% to 35.3%. The O_C_ fraction changed only slightly (22.4% to 23.4%).

The increased O_V_ contribution suggests that Ce/ZnO incorporation promotes defect formation in the In_2_O_3_ matrix, likely related to charge compensation associated with Ce valence transition. The coexistence of Ce^3+^/Ce^4+^ and the increased oxygen-vacancy concentration are expected to enhance surface oxygen adsorption and reaction activity, which is beneficial for ethanol sensing.

Overall, the XPS results are consistent with the proposed sensing mechanism and support the structural and compositional analyses obtained from XRD and EDS measurements.

### 3.2. Gas-Sensing Properties

The responses of gas sensors were measured in a gas chamber by controlling the temperature and gas concentration. To verify the gas-sensing performance, all the sensors were exposed to 20–100 ppm of ethanol gas in increments of 20 ppm.

**Figure 5:**
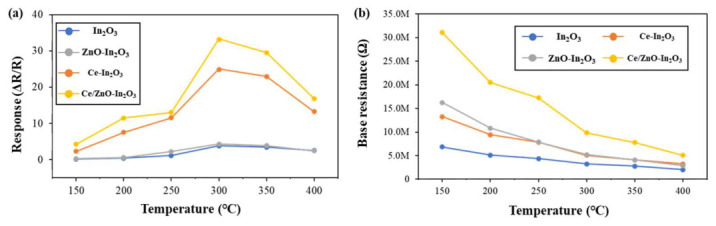
(**a**) Responses and (**b**) base resistances of In_2_O_3_, Ce-In_2_O_3_, ZnO-In_2_O_3_ and Ce/ZnO-In_2_O_3_ for 100 ppm of ethanol at various operating temperatures under 20% RH (defined at room temperature, 25 °C).

The temperature-dependent responses of the four distinct sensor materials to 100 ppm of ethanol gas are illustrated in Figure 5a. The responses exhibited by the four sensors under investigation generally followed a pattern. There was an increase in response with increasing temperature up to 300 °C and a decrease after 300 °C. This is due to the accelerated desorption of ethanol molecules and active oxygen species, which reduces surface reaction probability and electron transfer efficiency [44,45]. At an operating temperature lower than 150 °C, the ethanol molecules may not possess sufficient energy to overcome the energy barrier associated with adsorption, resulting in failure to adsorb on the surface of the sensor materials, In_2_O_3_ and ZnO [46]. All subsequent sensing tests were conducted at the optimal temperature of 300 °C. The corresponding temperature-dependent baseline resistances of the four sensors are presented in Figure 5b. At the same operating temperature, the baseline resistance differs significantly among the sensors, which can be attributed to intrinsic differences in charge carrier concentration, defect density, surface oxygen adsorption behavior, and heterojunction-induced potential barriers arising from Ce doping and ZnO incorporation. In addition, for each individual sensor, the baseline resistance varies with temperature due to thermally activated charge transport, temperature-dependent adsorption–desorption dynamics of oxygen species, and modulation of depletion layers at grain boundaries. These combined effects result in distinct resistance levels at identical temperatures and non-monotonic resistance variations with temperature for a given sensor material [47,48].

**Figure 6:**
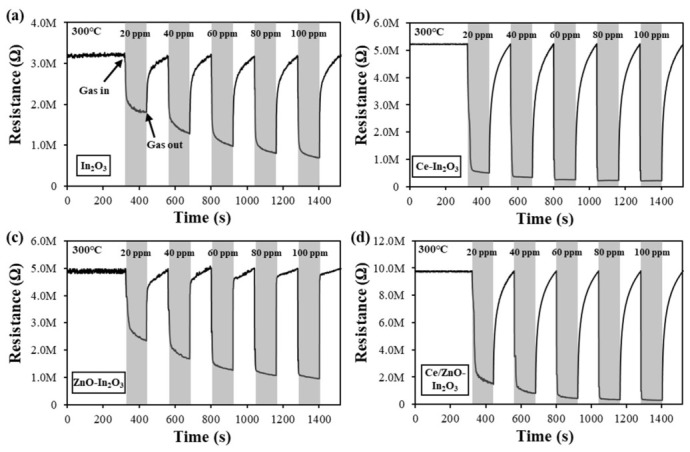
Resistance change graph of (**a**) In_2_O_3_, (**b**) Ce-In_2_O_3_, (**c**) ZnO-In_2_O_3_ and (**d**) Ce/ZnO-In_2_O_3_ for various ethanol gas concentrations at operating temperature 300 °C under 20% RH (defined at room temperature, 25 °C).

As shown in Figure 6, the dynamic response curves of the four distinct sensors are demonstrated in relation to ethanol. All sensors exhibited stable and reversible response and recovery behavior. The resistances of the sensors decreased when the ethanol gas was supplied and recovered to the initial value when the sensors were exposed to ambient air. This response to the reducing gas is consistent with the sensing behavior of n-type semiconductors. It is well established that both ZnO and In_2_O_3_ are n-type semiconductors. The magnitude of the observed resistance changes increased in proportion to the increase in ethanol gas concentration. The initial resistance of the pristine In_2_O_3_ NC was notably lower than the Ce and ZnO-incorporated In_2_O_3_ NCs. In_2_O_3_ is inherently an n-type semiconductor, with electrons provided by oxygen vacancies. However, when Ce is incorporated, the oxygen vacancy concentration is regulated depending on the oxidation state changes of Ce^3+^/Ce^4+^. Ce has been shown to increase lattice distortion and oxygen adsorption, leading to a decrease in free electron concentration and an increase in resistance [49]. Furthermore, when In_2_O_3_ and ZnO are combined, the disparity in work function between the two materials prompts the movement of electrons, thereby forming an n-n heterojunction at the interface. This process culminates in the formation of an electron depletion layer on the In_2_O_3_ side. This depletion layer imposes restrictions on the conduction path, thereby increasing the initial electrical resistance of the entire structure [50]. Consequently, the Ce/ZnO-In_2_O_3_ composite, where both the Ce and the ZnO bonding effect act in tandem, manifests an elevated initial resistance in comparison to pure In_2_O_3_.

**Figure 7:**
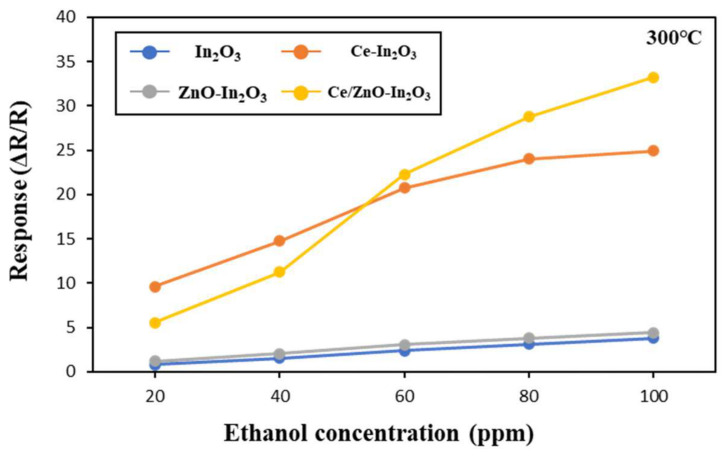
Responses of In_2_O_3_, Ce-In_2_O_3_, ZnO-In_2_O_3_ and Ce/ZnO-In_2_O_3_ for various ethanol gas concentrations at operating temperature 300 °C under 20% RH (defined at room temperature, 25 °C).

The test of the dynamic sensing characteristics of four distinct gas sensors constructed using In_2_O_3_, Ce-In_2_O_3_, ZnO-In_2_O_3_, and Ce/ZnO-In_2_O_3_ in response to ethanol gas is presented. As demonstrated in Figure 7, an examination of gas sensors was conducted to ascertain their response to varying concentrations of ethanol, ranging from 20 to 100 ppm at 300 °C. The Ce/ZnO-In_2_O_3_ sensors exhibited the most pronounced response at a concentration of 100 ppm, with the response of 33.26. The incorporated samples, namely Ce-In_2_O_3_ and Ce/ZnO-In_2_O_3_, exhibited a higher response than the pristine In_2_O_3_. Additionally, the ZnO-In_2_O_3_ demonstrates a marginally elevated response in comparison to the In_2_O_3_ gas sensor.

The response and recovery times of all four sensors were extracted from the dynamic curves in Figure 6 using the standard 90%/10% criterion, defined as the times to reach 90% and return to within 10% of the total resistance change, respectively. The values at 100 ppm ethanol and 300 °C are summarized in Table 1. Ce incorporation shortens the response time due to the additional oxygen vacancies serving as fast adsorption sites, while the ZnO-In_2_O_3_ heterojunction slightly prolongs both response and recovery due to the interfacial potential barrier. The Ce/ZnO-In_2_O_3_ composite shows a balanced kinetic performance with the highest response magnitude, demonstrating the synergy between Ce doping and heterojunction formation.

**Figure 8:**
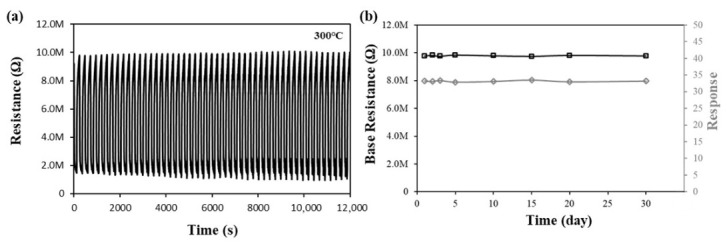
(**a**) Repeatability resistance graph of Ce/ZnO-In_2_O_3_ for 20 ppm ethanol. (**b**) Long-term stability base resistance and response graph of Ce/ZnO-In_2_O_3_ for 100 ppm ethanol. The sensor was operated at 300 °C, and the relative humidity (20% RH) was controlled at room temperature (25 °C).

Ensuring the reliability of measurement values and enabling the commercialization of the sensor is contingent upon the consistency of gas reactions in sensor applications across repeated and long-term stable tests. Those tests for Ce/ZnO-In_2_O_3_ were performed about ethanol gas. As demonstrated in Figure 8a, the Ce/ZnO-In_2_O_3_ demonstrated a response of approximately 6.5 for the ethanol concentration of 20 ppm, repeated 100 times. Also, Figure 8b shows a stable base resistance and response for a month with 100 ppm ethanol. The limit of detection (LoD) for ethanol gas for Ce/ZnO-In_2_O_3_ was calculated using the method of noise of the sensor and slope for response, as outlined by the International Union of Pure and Applied Chemistry (IUPAC) [51]. The baseline noise of the Ce/ZnO-In_2_O_3_ sensor was characterized by recording the sensor resistance in clean synthetic air (1000 sccm, 300 °C, 20% RH) for 30 min at 1 s sampling intervals (*n* = 1800). From this baseline time-series, the standard deviation S and the root-mean-square deviation N were calculated, and the RMS noise was computed according to Equation (2):

(2) $${RMS}_{noise}=\sqrt{\frac{S^{2}}{N}}$$

The sensor response slope was obtained via linear regression of the response (ΔR/R) versus ethanol concentration over 20–100 ppm, yielding a slope of 36.4 ppm^−1^ (RMS_noise_ = ~9.84). Calculations revealed that the LoD of this sensor for ethanol gas is approximately 0.8 ppm, as calculated from Equation (3):

(3) $$Limit\;of\;Detection\;(LOD)=3\frac{{RMS}_{noise}}{Slope}$$

To further contextualize the sensing performance, Table 2 summarizes recently reported ethanol-sensing performance of ZnO-based, In_2_O_3_-based, Ce-modified, and ZnO/In_2_O_3_ heterostructure sensors. The Ce/ZnO-In_2_O_3_ sensor achieves a response of 33.2 (ΔR/R) at 100 ppm ethanol with a detection limit of 0.8 ppm, competitive with the best reported ZnO/In_2_O_3_ heterostructure sensors and Ce-modified oxide sensors. More importantly, the combined strategy simultaneously provides humidity tolerance (~77% response retention at 80% RH), long-term stability (~1 month), and a nearly two-fold enhancement in surface oxygen vacancy density-performance metrics that are rarely reported together in the prior literature.

**Figure 9:**
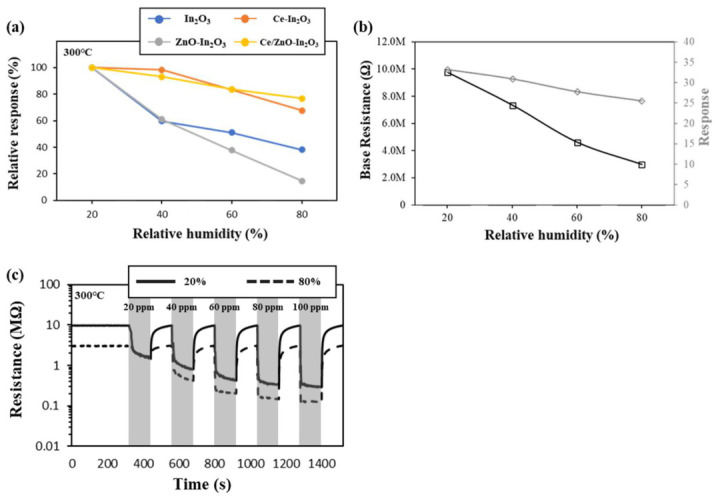
(**a**) Relative response variation in the sensors as a function of relative humidity (20–80% RH) toward 100 ppm ethanol at an operating temperature of 300 °C. (**b**) Dependence of base resistance and gas response of the Ce/ZnO-In_2_O_3_ sensor on relative humidity (20–80% RH) under the same conditions. (**c**) Dynamic resistance changes in the Ce/ZnO-In_2_O_3_ sensor at 20% and 80% RH upon exposure to ethanol at an operating temperature of 300 °C. Relative humidity was controlled at room temperature (25 °C), whereas the sensor operating temperature was 300 °C.

In the context of practical applications, humidity must be considered a significant factor in the performance of MOS sensors. In this study, the Ce was employed to separate the water in air from the sensing materials, thereby enhancing the anti-humidity properties of the In_2_O_3_ sensors. As demonstrated in Figure 9a, the relative response values of all sensors and the normalized response for its initial response at relative humidity of 20% RH as the standard, respectively, exhibit a consistent decrease with increasing humidity to 100 ppm ethanol at 300 °C. However, the Ce-incorporated sensors demonstrate relatively high response values. In order to ascertain the effect of Ce on the anti-humidity of the gas sensors, the relative response variation of each sensor was normalized using its initial response at a humidity of 20% relative humidity as the standard at 300 °C. At 80% RH, the Ce/ZnO-In_2_O_3_ sensor demonstrates the highest response retention of 76.84%, while the In_2_O_3_ sensor exhibits a response retention of only 38.09%. This enhancement in response can be predominantly attributed to the diminished impact of humidity, a phenomenon that is facilitated by the protective effect of Ce. Rare earth (Pr, Ce, La, etc.) has been shown to regulate surface oxygen species through redox pairs (e.g., Ce^3+^/Ce^4+^), readjust oxygen vacancy/electron density, and modify surface acidity and adsorption energy. This has been observed to reduce humidity sensitivity or, in some cases, result in a trade-off between sensitivity and humidity [52]. The graph showing the base resistance and response for 100 ppm ethanol of Ce/ZnO-In_2_O_3_ with different relative humidity at 300 °C is shown in Figure 9b. Also, Figure 9c shows the resistance changes of Ce-doped ZnO-In_2_O_3_ according to ethanol concentration at 20% and 80% RH (defined at 25 °C), with the sensor operated at 300 °C. The improved humidity tolerance of Ce-containing sensors arises from multiple cooperating mechanisms, all traceable to the mixed-valence Ce^3+^/Ce^4+^ surface chemistry directly confirmed by XPS. Catalytic turnover of surface hydroxyl groups by Ce^3+^/Ce^4+^ redox cycling, 2OH^−^ + Ce^4+^ → H_2_O ↑ + O^2−^ lattice + Ce^3+^, regenerates lattice oxygen and restores active O^−^ adsorption sites otherwise blocked by water adsorption. Preferential Lewis-acid binding of ethanol over water on Ce^3+^ sites maintains ethanol sensitivity. Buffering of humidity-induced electron injection by the Ce redox pair preserves the baseline resistance dynamic range. There is intrinsically lower water-adsorption energy on CeO_x_ surfaces compared to In_2_O_3_/ZnO [52]. These effects collectively explain the greater than two-fold improvement in response retention at 80% RH in Ce-containing sensors relative to pristine In_2_O_3_. The enhanced O_V_ fraction directly evidenced by the O 1s XPS further supports this mechanism, as oxygen vacancies associated with Ce^3+^ sites compete effectively with water molecules for surface adsorption sites.

**Figure 10:**
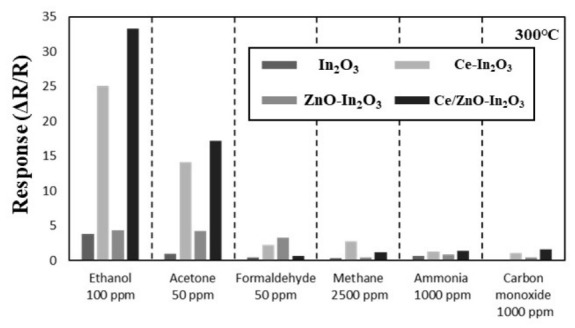
Selectivity bar graph of In_2_O_3_, Ce-In_2_O_3_, ZnO-In_2_O_3_ and Ce/ZnO-In_2_O_3_ for 100 ppm ethanol, 50 ppm acetone, 50 ppm formaldehyde, 2500 ppm methane, 1000 ppm ammonia and 1000 ppm carbon monoxide at operating temperature 300 °C under 20% RH (defined at room temperature, 25 °C).

**Figure 11:**
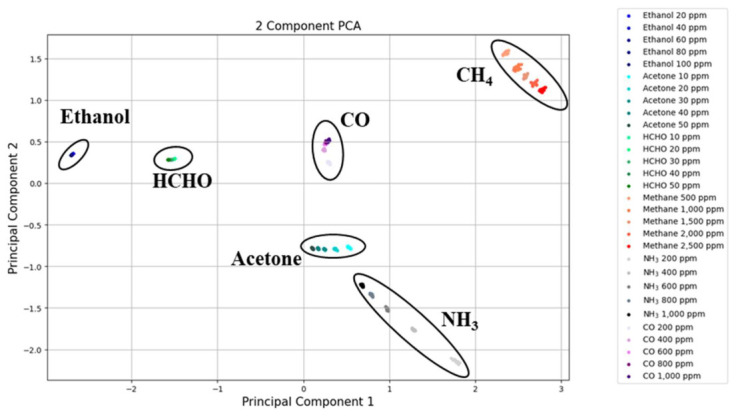
PCA results of In_2_O_3_ sensors with Ce and ZnO incorporation for ethanol, acetone, formaldehyde, methane, ammonia and carbon monoxide.

As demonstrated in numerous preceding studies, ZnO and In_2_O_3_ have been extensively utilized as model gas sensor materials. However, when utilized in their pure form, these materials exhibit limitations in that they do not adequately ensure selectivity for specific gases. Consequently, active research has been conducted to enhance sensitivity and selectivity through various incorporation and heterojunction formations [3,9,16,41]. Figure 10 shows the selectivity for ethanol through bar graphs; Ce-incorporated sensors, especially Ce/ZnO-In_2_O_3_, exhibited higher responses to ethanol than other gases. The target gas concentrations used in the selectivity test (ethanol 100 ppm; acetone and formaldehyde 50 ppm; methane 2500 ppm; ammonia and CO 1000 ppm) were selected based on their respective occupational exposure thresholds and the measurable response ranges of each gas on In_2_O_3_-based sensors. Lower-response gases (CH_4_, NH_3_, CO) were tested at concentrations near their IDLH or LEL-fraction alarm levels to provide measurable responses above the noise floor, while alcohol and aldehyde gases were tested at lower concentrations representative of typical indoor/industrial exposure. However, as the response to ethanol was improved by Ce, responses to other gases had also been improved compared to pristine In_2_O_3_. Also, this higher response to ethanol was exhibited at 300 °C. This phenomenon can be attributed to the enhanced dissociation rate of ethanol at the surface of In_2_O_3_ at this temperature. It is important to note that the ethanol selectivity reported here is maximized at the 300 °C optimal operating temperature, at which the Ce^3+^/Ce^4+^-catalyzed dehydrogenation pathway is kinetically most favorable. At lower operating temperatures (150–250 °C), the optimal response temperatures of other gases such as formaldehyde become competitive with ethanol, partially reducing selectivity; at higher temperatures (>350 °C), the responses of all gases decrease. However, it is possible that the sensor may exhibit a higher response to other gases at different temperatures. In consideration of these factors, a principal component analysis (PCA) was employed to analyze the results. PCA is a statistical technique that summarizes high-dimensional data through dimension reduction and information visualization while maintaining key trends and patterns [53,54]. This analysis was conducted to more clearly confirm the selectivity of ethanol gas. The experiment utilized four gas sensors, In_2_O_3_, Ce-In_2_O_3_, ZnO-In_2_O_3_, and Ce/ZnO-In_2_O_3_, on the aforementioned outcomes. Among these, In_2_O_3_ and Ce/ZnO-In_2_O_3_ were each applied in four additional types for each measurement temperature condition from 150 °C to 300 °C, resulting in a total of 10 sensors being utilized. As illustrated in Figure 11, the PCA results for gas responses are evident. The input data matrix was constructed from the response values (ΔR/R, Equation (1)) of ten sensor instances (pristine In_2_O_3_ and Ce/ZnO-In_2_O_3_, each measured at 150, 200, 250, and 300 °C; Ce-In_2_O_3_ and ZnO-In_2_O_3_, each measured at 300 °C) to six target gases (ethanol, acetone, formaldehyde, methane, ammonia, and carbon monoxide) at the concentrations specified above. Each response feature was standardized using z-score normalization (zero mean and unit variance) prior to PCA to prevent features with larger response magnitudes from dominating the principal components. PCA was performed with n components = 2 using full singular-value decomposition. The first two principal components captured 75% (PC1) and 25% (PC2) of the total variance, respectively, accounting for 100% of the variance in the normalized dataset. The results of this study were calculated using the standard PCA procedure in Python (3.12.13, Google Colab), which explained 100% of the total variation. The input variables for PCA are the gas detection response values defined in Equations (1) and (2), based on data for six types of gases for each of the ten sensor types. Consequently, no instances of overlap were detected for any gas or concentration, and the directionality of each gas concentration change was distinctly discernible. This result demonstrates the better selectivity.

In the context of the gas sensor, the surface of the material is exposed to the air atmosphere, resulting in the absorption of oxygen molecules. These molecules subsequently capture electrons from the sensing material, undergoing a transformation into chemisorbed oxygen. Subsequently, the electron concentration of the sensitive body will undergo a decrease, thereby forming electron depletion layers of considerable thickness on the material surface. This phenomenon will culminate in the development of a high resistance state within the gas sensor [4,55]. However, when the gas sensor is exposed to an ethanol atmosphere, a series of chemical reactions occur between chemisorbed oxygen and ethanol molecules. These reactions result in the release of electrons back to the sensitive body, leading to a decrease in the gas sensor resistance [56]. The dominant adsorbed oxygen species depends on the surface temperature [47,48]: O_2_^−^ (superoxide) dominates in the 100–200 °C range, O^−^ (atomic anion) becomes dominant in the 200–400 °C range, and O^2−^ (lattice oxygen) predominates above 400 °C. At the operating temperature of 300 °C employed in this study, O^−^ is the dominant chemisorbed oxygen species. The aforementioned processes can be summarized as follows:

(4) $$O_{2}\left(g\right)+e^{-}\to O_{2}^{-}\;(100\;{}^{\circ}C<T<200\;{}^{\circ}C)$$

(5) $$O_{2}^{-}+e^{-}\to {2O}^{-}\;(200\;{}^{\circ}C<T<400\;{}^{\circ}C)$$

(6) $$C_{2}H_{5}OH\left(g\right)\to C_{2}H_{5}OH\left(ads\right)$$

(7) $$C_{2}H_{5}OH\left(ads\right)+{6O}^{-}\to 2CO_{2}+3H_{2}O+{6e}^{-}$$

**Figure 12:**
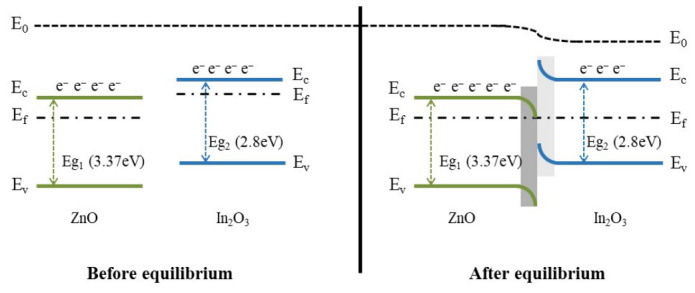
Energy band diagram of ZnO-In_2_O_3_.

The synergistic effect of ZnO-In_2_O_3_ heterojunctions is attributed to the enhanced performance in the ethanol reaction. It has been established that electron transfer occurs from the conduction band of In_2_O_3_ (3.5 eV) to that of ZnO (4.45 eV) due to the disparate work functions of these two compounds. This process results in Fermi level equilibration, as shown in Figure 12. The redistribution of charge leads to the formation of an electron accumulation layer on the ZnO side and an electron depletion layer on the In_2_O_3_ side at the heterojunction interface. For ZnO-In_2_O_3_, the presence of a ZnO electron accumulation layer leads to the creation of a significantly higher number of active sites capable of adsorbing oxygen. In contrast, In_2_O_3_ with an electron depletion layer has been observed to elicit a discernible change in resistance when the analyte reacts with adsorbed oxygen. The sensing mechanism is regulated by both the accumulation and depletion layers in the heterojunctions [21,57].

The incorporation of Ce into the In_2_O_3_ lattice induces a distortion of the crystal structure, thereby generating additional defects and active sites. This is directly evidenced by the Ce 3d XPS analysis (Figure 4a), which reveals a Ce^3+^/(Ce^3+^ + Ce^4+^) ratio of 32.4% in the Ce/ZnO-In_2_O_3_ composite, and by the O 1s XPS (Figure 4b,c), which shows a near-doubling of the oxygen vacancy density (41.3% vs. 22.5% in pristine In_2_O_3_). The observed lattice asymmetry has been shown to induce alterations in the local electron distribution and the bandgap [58]. The changing chemical valence state of Ce^3+^, Ce^4+^ could be another significant reason for the enhanced gas-sensing performance. The process can be categorized into two distinct aspects. The cerium typically exists in the form of Ce^4+^, but a proportion of Ce^4+^ ions can undergo conversion to Ce^3+^, resulting in the release of oxygen vacancies. The oxygen vacancies are a significant defect that can enhance sensitivity [4,59]. The Ce^3+^ state has the capacity to absorb electrons from oxygen, undergoing a transformation into the Ce^4+^ state. This process enhances the electrical resistance of the materials. When Ce^4+^ is exposed to a reducing gas, it undergoes a reduction to Ce^3+^, thereby increasing the degree of resistance drop [37]. Beyond this general redox behavior, the preferential enhancement of ethanol sensing over other reducing gases by Ce incorporation arises from the specific molecular-level interaction between ethanol and the Ce^3+^/Ce^4+^ Lewis-acid sites. Ce^3+^, being a moderately hard Lewis acid, strongly coordinates with the oxygen lone pair of the hydroxyl group of ethanol, lowering the O-H bond activation energy and initiating dehydrogenation to an adsorbed ethoxide species. Subsequent β-hydride elimination yields adsorbed acetaldehyde (CH_3_CHO), which is further oxidized by surface O^−^ species to CO_2_ and H_2_O, releasing a total of six electrons per ethanol molecule (Equation (7)) and amplifying the resistance change. In contrast, other reducing analytes either lack a polar O-H group for Lewis-acid coordination (e.g., CH_4_, CO), have stronger C-H or C-C bonds requiring higher activation temperatures (CH_4_ typically requires T > 400 °C), or undergo single-step oxidation releasing fewer electrons per molecule. Consequently, while Ce incorporation generally increases surface oxygen availability for all reducing gases, the multi-step, hydride-transfer-based oxidation pathway unique to ethanol receives a disproportionately large catalytic acceleration from the Ce^3+^/Ce^4+^ sites, resulting in the pronounced ethanol selectivity observed in Figure 10 and the clear clustering of the ethanol response vectors in the PCA analysis (Figure 11).

## 4. Conclusions

In this study, Ce/ZnO-In_2_O_3_ nanocube composites were successfully synthesized and demonstrated superior ethanol gas-sensing performance compared to pristine In_2_O_3_. The optimized sensor demonstrated a response of 33.2 at 100 ppm, with a detection limit of 0.8 ppm. It exhibited fast and reversible switching behavior, as well as stable repeatability over 100 cycles. The synergistic effects of Ce and ZnO heterojunction formation have been demonstrated to improve oxygen adsorption, electron transport, and surface defect engineering, thereby enhancing sensitivity and humidity resistance. PCA analysis further validated the enhanced selectivity toward ethanol. Collectively, these findings suggest that Ce/ZnO-In_2_O_3_ is a promising candidate for high-performance ethanol detection, with potential applicability in environmental monitoring, food safety, and industrial safety systems. Although the optimal operating temperature of 300 °C identified in this study is higher than typically desired for battery-powered portable devices, practical implementation remains feasible through integration with MEMS-based micro-heaters. Such systems have been reported to sustain operating temperatures around 300 °C with low power consumption (10–50 mW) and reliable long-term stability [12]. Accordingly, the associated high operating temperature can be effectively mitigated and does not preclude practical deployment.

Moreover, the Ce-ZnO heterojunction synergy observed in this work enhances the ethanol sensing response over a broad temperature range (150–400 °C). This behavior indicates that the proposed design strategy retains its effectiveness even at reduced operating temperatures. While additional optimization such as catalytic functionalization or increased surface area through nanostructure engineering may be required to achieve optimal performance at lower temperatures, the present results support the feasibility of extending this approach toward lower-temperature operation. Further investigations aimed at achieving efficient sensor operation at reduced temperatures are currently in progress and will be reported shortly.

## Figures and Tables

**Table 1:** Response (τ_res_) and recovery (τ_rec_) times of the four sensors toward 100 ppm ethanol at 300 °C.

Sensor	τ_res_ (s)	τ_rec_ (s)
In_2_O_3_	40	180
Ce-In_2_O_3_	28	100
ZnO-In_2_O_3_	35	120
Ce/ZnO-In_2_O_3_	25	90

**Table 2:** Comparison of ethanol-sensing performance of recently reported ZnO/In_2_O_3_ and ZnO-, In_2_O_3_- and Ce-modified heterostructure sensors.

Material	Mechanism	Target	Conc.	Response	T (°C)	LOD	Ref.
ZnO microspheres	Pristine ZnO	Ethanol	100	~8.3	340	-	[7]
In_2_O_3_ nanobelts	Pristine In_2_O_3_	Ethanol	100	~5.1	250	-	[5]
ZnO-In_2_O_3_ hierarchical	n-n heterojunction	Ethanol	100	~28.4	260	~1.5	[31]
In_2_O_3_-ZnO mesoporous	n-n heterojunction	Ethanol	100	~22.7	280	-	[42]
ZnO@ In_2_O_3_@ZnO	n-n heterojunction	Ethanol	100	~36.1	250	~0.5	[30]
Ce-In_2_O_3_ nanospheres	Ce doping	Methanol	100	~18.3	220	~2.0	[44]
Ce-In_2_O_3_ nanosheet	Ce doping	Isopropanol	100	~31	220	~1.2	[43]
Ce/ZnO-In_2_O_3_ (This work)	Ce + n-n heterojunction	Ethanol	100	33.2	300	0.8	-

## Data Availability

All data are available from the corresponding author upon request.
